# Ramosetron versus ondansetron for postoperative nausea and vomiting in strabismus surgery patients

**DOI:** 10.1186/s12871-016-0210-5

**Published:** 2016-07-22

**Authors:** Jin Joo, Shinhye Park, Hue Jung Park, Sun Young Shin

**Affiliations:** 1 Department of Anesthesiology and Pain Medicine, Seoul St. Mary’s Hospital, College of Medicine, The Catholic University of Korea, Seoul, Korea; 2 Department of Ophthalmology, Seoul St. Mary’s Hospital, College of Medicine, The Catholic University of Korea, Seoul, Korea

**Keywords:** Postoperative nausea, Strabismus surgery, Ramosetron, Ondansetron

## Abstract

**Background:**

Postoperative nausea and vomiting (PONV) is one of the most common adverse outcomes after strabismus surgery. The primary outcome of this prospective, randomized, double-blind study was to compare the incidences of nausea or vomiting, and patient satisfaction of ondansetron and ramosetron after strabismus surgery under general anesthesia. The secondary outcome was to investigate whether the number of involved extraocular muscles (EOMs) in strabismus surgery was related to PONV.

**Methods:**

One hundred and five patients (aged 18–60 years) undergoing strabismus surgery were allocated randomly to one of the three groups: placebo, ondansetron, or ramosetron. Patients received 2 ml placebo, 4 mg ondansetron, or 0.3 mg ramosetron at the end of surgery. Each of the three groups was subdivided into two subgroups according to the number of EOMs involved in the surgery: subgroup S, single-muscle correction; subgroup M, multiple-muscle correction. The incidences of nausea or vomiting, and patient satisfaction at 2, 24 and 48 h after surgery were analyzed as primary outcome. With regard to subgroups S and M in the placebo, ondansetron and ramosetron groups, incidences of nausea or vomiting, and patient satisfaction at 2, 24 and 48 h after surgery were analyzed as seconadary outcome.

**Results:**

The incidence of nausea was significantly lower in the ramosetron group at 2 h (9.4 %) than in the placebo (45.2 %) and ondansetron (34.7 %) groups (*P* < 0.05). The incidence of nausea was also significantly lower in the ramosetron group at 24 h than in the other groups (*P* < 0.05). Patients in the ramosetron group were more satisfied at 2 h (8.11 ± 0.98) and 24 h (8.50 ± 0.67) after surgery than those in the other groups (*P* < 0.05). With regard to subgroups S and M in the placebo, ondansetron and ramosetron groups, there were no significant differences in either the incidence of nausea or patient satisfaction.

**Conclusion:**

Ramosetron has superior antiemetic activity to ondansetron in adult strabismus surgery patients. The number of EOMs involved in strabismus surgery was not related to the incidence of PONV.

**Trial registration:**

Clinical Research Information Service (CRiS) Identifier: KCT0000688. Date of registration: 27 February 2013.

## Background

Postoperative nausea and vomiting (PONV) is one of the most common adverse outcomes after strabismus surgery under general anesthesia, with an incidence of 37–80 % [1–4]. PONV may delay discharge from hospital or cause unexpected hospitalization in severe cases not only that it lowers patient’s satisfaction after surgery.

There are several known methods for PONV prophylaxis. Serotonin (5-HT_3_) receptor antagonists are recommended as the first-line regimen for PONV prophylaxis [5]. Ondansetron, the first 5-HT_3_ receptor antagonist used clinically for the prevention and management of PONV, acts less selectively on the 5-HT_3_ receptor than other 5-HT_3_ antagonists. Systemic review has revealed that the prophylactic effect of ondansetron on nausea is questionable although it has fine prophylactic effect on vomiting [6, 7]. Ramosetron, a newer 5-HT_3_ antagonist, has higher affinity to the 5-HT_3_ receptor and longer duration of action, and has a similar or greater prophylactic effect on PONV compared with older 5-HT_3_–receptor antagonists such as granisetron and ondansetron [8–10].

Traction of extraocular muscles (EOMs) is one of the factors that trigger PONV after strabismus surgery, which induces the oculoemetic reflex [11]. We hypothesized that more PONV would occur as the number of EOMs involved in strabismus surgery increases, causing more muscle traction. To our knowledge, no previous reports of the relationship between the numbers of involved EOMs and PONV have been published.

Thus, we primarily designed this prospective, randomized, double-blind study to compare the incidences of nausea or vomiting, and patient satisfaction of ondansetron and ramosetron after strabismus surgery under general anesthesia in patients aged > 18 years. The secondary outcome was to investigate whether the number of involved extraocular muscles (EOMs) in strabismus surgery was related to PONV.

## Methods

### Study population

One hundred twenty patients aged between 18 and 60 years, ASA physical status I and II, and scheduled for strabismus surgery under general anesthesia were enrolled from April 2011 to April 2012 in this prospective, randomized, double-blind clinical study. Patients who took any analgesics, steroids or antiemetics 24 h before surgery, or who had gastrointestinal diseases were excluded.

Recruited patients were allocated randomly to receive one of the following three drugs at the end of surgery: intravenous (IV) placebo (normal saline), ondansetron (4 mg), or ramosetron (0.3 mg) [12, 13]. A computerized randomization list was generated, and identical syringes (2 ml total) containing each drug were prepared by personnel not involved in the study, according to the list.

### Study protocol

All patients were allowed to take solid food up to 8 h before surgery and water up to 2 h before surgery. No premedication was administered to the patients. Electrocardiogram, non-invasive blood pressure, heart rate, and pulse oximetry were measured continuously at 5-min intervals, starting from the time of arrival in the operating room. General anesthesia was induced with 2 mg/kg propofol and 0.6 mg/kg rocuronium. After tracheal intubation, anesthesia was maintained with 1.5–2 vol.% sevoflurane, medical air in oxygen [fraction of inspired O_2_ (FiO_2_) = 0.5], and continuously infused IV remifentanil 0.05–0.1 μg/kg/min, keeping end-tidal CO_2_ between 35 and 40 mmHg throughout the surgery. Ringer’s lactate solution was administered at 6–8 ml/kg/h during surgery. The nasopharyngeal temperature was monitored and maintained at 36 ± 1 °C throughout surgery using a warming pad. The types of strabismus surgery included were unilateral rectus muscle recession, unilateral rectus muscle resection, bilateral rectus muscle recession, and bilateral rectus muscle resection. At the end of the surgical procedure, sevoflurane and remifentanil administration were discontinued. IV 0.2 mg/kg pyridostigmine and 0.008 mg/kg glycopyrrolate were administered for reversal of muscle relaxation, along with the assigned study drug. The trachea was extubated when spontaneous respiration of the patient was adequate.

After the operation, the patients were transferred to the postanesthesia care unit. Every episode of nausea or vomiting was recorded during the first 2 h after surgery, and the presence of nausea at 2 h after surgery was asked by a member of the nursing staff who was blinded to the study drug used. She also filled out the data table including the followings: presence of nausea and vomiting, use of rescue antiemetics, patient’s satisfaction, degree of pain, use of rescue analgesics, and presence of adverse effects of the study drugs, such as headache, dizziness, or drowsiness. The patients were asked to grade their satisfaction from 0 to 10 (VRS, verbal rating scale), where 0 represents total dissatisfaction and 10 represents the most satisfactory subjective answer. The degree of pain was recorded using a visual rating scale (VRS) with options from 0 (not painful at all) to 10 (extremely painful).

If VRS score was > 3 for > 5 min, 30 mg IV ketorolac was administered for rescue analgesia. If patients complained of severe pain (VRS > 7), 1 μg/kg fentanyl was administered. When patients complained of nausea or vomitted, 10-mg IV metoclopramide was administered as a rescue antiemetic.

Patients were discharged from the hospital unless they showed severe complications of anesthesia, such as fever or desaturation due to laryngospasm or atelectasis. At 24 and 48 h after surgery, a resident of the Department of Ophthalmology who was also blinded to the study drug used completed the data table via telephone call.

After completion of data collection, patients were subdivided into two groups according to the number of EOMs involved in the surgery: subgroup S, single-muscle correction; and subgroup M, multiple-muscle correction.

### Statistical analyses

To estimate the required sample size, a power analysis was used. We aimed for an 80 % probability (β = 0.2) of detecting a 50 % difference with a significance level (α) of 0.05, based on the incidence of PONV being 50 % in the placebo group and 20 % in the ramosetron group from our pilot study; thus 25 patients per group were required. To account for exclusion of some patients, we enrolled 35 patients in each group. Statistical analysis was performed using the SPSS, version 18.0 (SPSS, Chicago, IL, USA). Demographic data were analyzed by Student’s *t*-test. Differences in the incidence of nausea and vomiting, and use of rescue antiemetics were analyzed by *χ* ^2^ test. Patient’s satisfaction and pain were compared using one-way ANOVA with *post hoc* Bonferroni correction for multiple comparisons; data are presented as mean ± SD. Intention-to-treat (ITT) analysis was conducted using last-observation-carried-forward (LOCF) imputation for missing values. A value of *P* < 0.05 was considered to indicate statistical significance.

## Results

**Fig. 1:**
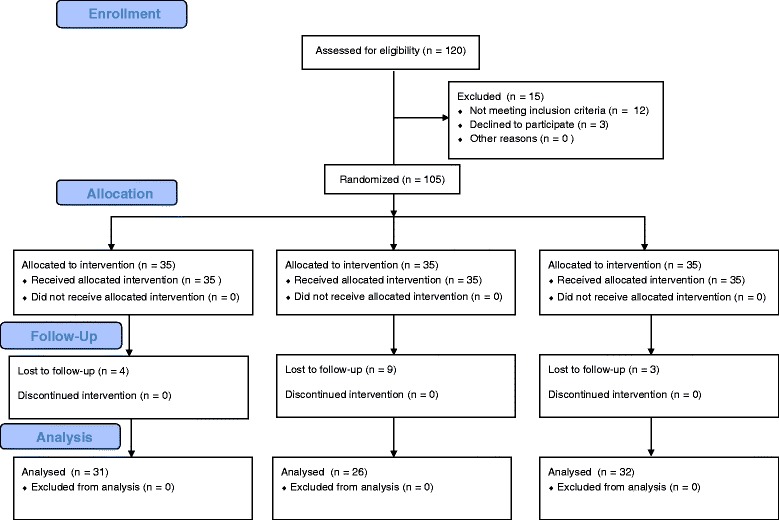
Flow diagram of the study

Among 120 patients recruited, 12 patients were excluded because they did not meet inclusion criteria; they showed ASA physical status >2. Another three patients were excluded because they did not agree to participate. Hence, 105 were randomly allocated to three groups. Among 16 follow-up loss patients, three patients and 13 patients did not respond to 24 h and 48 h telephone call, respectively (Fig. 1). Since persistent prophylactic effect on PONV was in concern, all the follow-up loss patients were excluded. There were no significant differences in patients’ characteristics, Apfel’s four risk factors for PONV (female gender, nonsmoking, prior history of motion sickness or PONV, and the use of intraoperative opioids) [14], and the duration of surgery and anesthesia among the groups (Table 1).

**Table 1:** Patients’ characteristics and clinical data

	Placebo	Ondansetron	Ramosetron
	(*n* = 31)	(*n* = 26)	(*n* = 32)
Age, years	34.10 ± 15.14	35.69 ± 17.13	34.13 ± 13.66
Weight, kg	61.38 ± 9.50	65.32 ± 10.63	59.64 ± 10.39
Height, cm	163.82 ± 10.79	167.70 ± 9.63	165.49 ± 10.33
Sex, M/F	14/17	14/12	13/19
PONV Hx., n (%)	2 (6.45)	2 (7.69)	2 (6.25)
Motion sickness, n (%)	12 (38.71)	10 (38.46)	13 (40.63)
Non-smoker, n (%)	20 (64.52)	17 (65.38)	21 (65.63)
Duration of surgery, min	23.90 ± 3.82	24.32 ± 3.72	24.05 ± 2.36
Duration of anesthesia, min	35.24 ± 7.92	35.95 ± 4.26	36.64 ± 4.97

Data are means ± SD or numbers. *F* female, *M* male

**Fig. 2:**
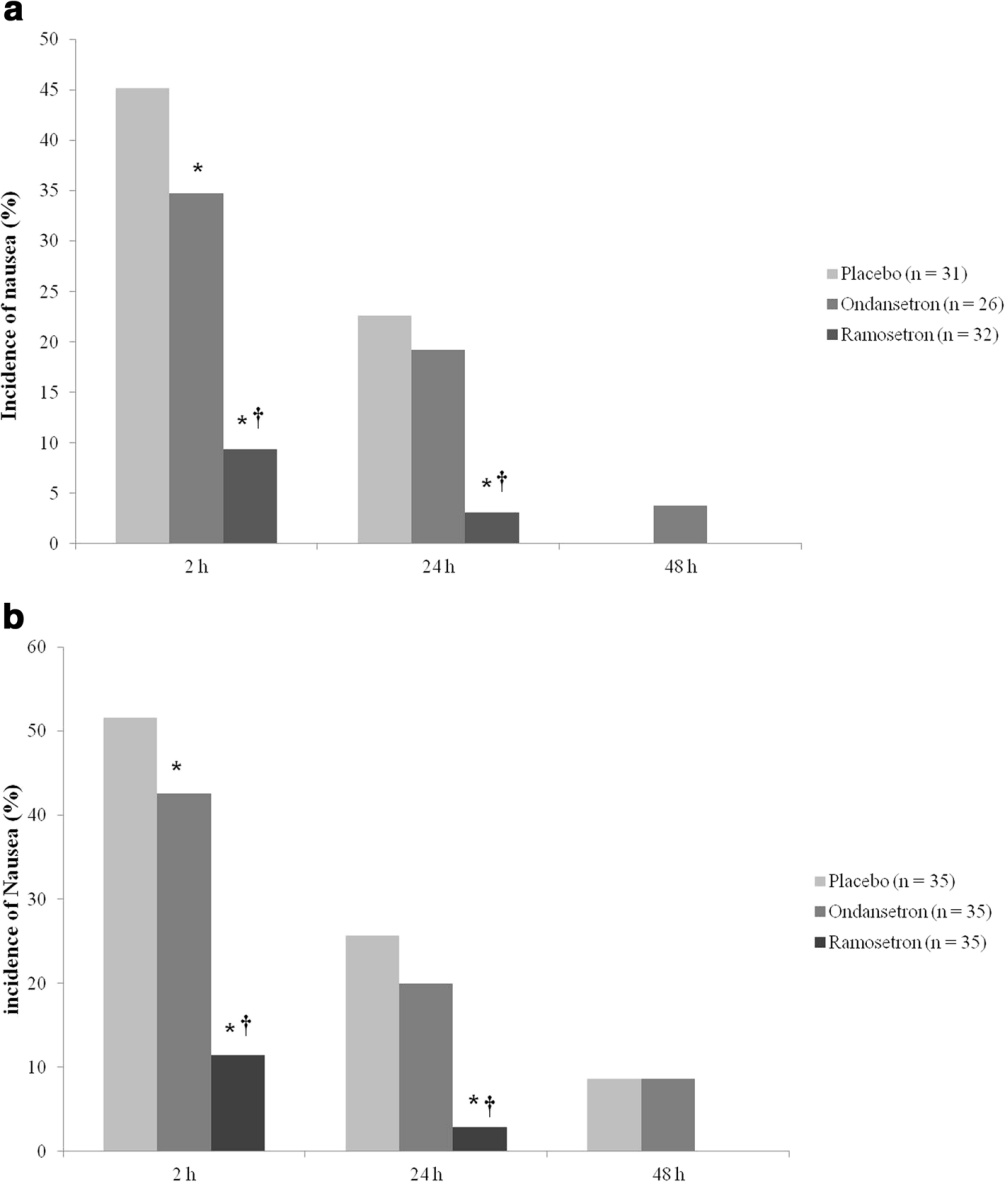
Incidence of nausea. Per- protocol analysis (**a**) and intention-to-treat analysis (**b**). *P < 0.05 versus placebo group, †*P* < 0.05 versus ondansetron group

The total incidences of nausea were 29.2 % (26/89) at 2 h, 14.6 % (13/89) at 24 h, and 1.1 % (1/89) at 48 h after surgery. The incidence of nausea was significantly lower in the ramosetron group at 2 h (9.4 %) than in the placebo (45.2 %) and ondansetron (34.6 %) groups (*P* < 0.05). The incidence of nausea was also significantly lower in the ramosetron group at 24 h (3.1 %) than in the placebo (22.6 %) and ondansetron (19.2 %) groups (*P* < 0.05). There was no significant difference in the incidence of nausea at 48 h after surgery. The ondansetron group, comparing with the placebo group, showed significantly lower incidence of nausea only at 2 h after surgery (Fig. 2a). The ITT analysis using LOCF imputation for missing values revealed similar results (Fig. 2b). No patient showed vomiting after surgery. The incidence of rescue antiemetic use was identical significantly lower in the ramosetron group than in the other groups. No patients required fentanyl as rescue analgesics (Table 2).

**Table 2:** Use of rescue analgesics and antiemetics in postanestheia unit

	Placebo	Ondansetron	Ramosetron
	(*n* = 31)	(*n* = 26)	(*n* = 32)
Use of rescue analgesics, n (%)	17 (54.84)	14 (53.85)	18 (56.25)
Use of rescue antiemetics, n (%)	14 (45.2)	9 (34.6)*	3 (9.4)*†

*P < 0.05 versus placebo group, †*P* < 0.05 versus ondansetron group

**Fig. 3:**
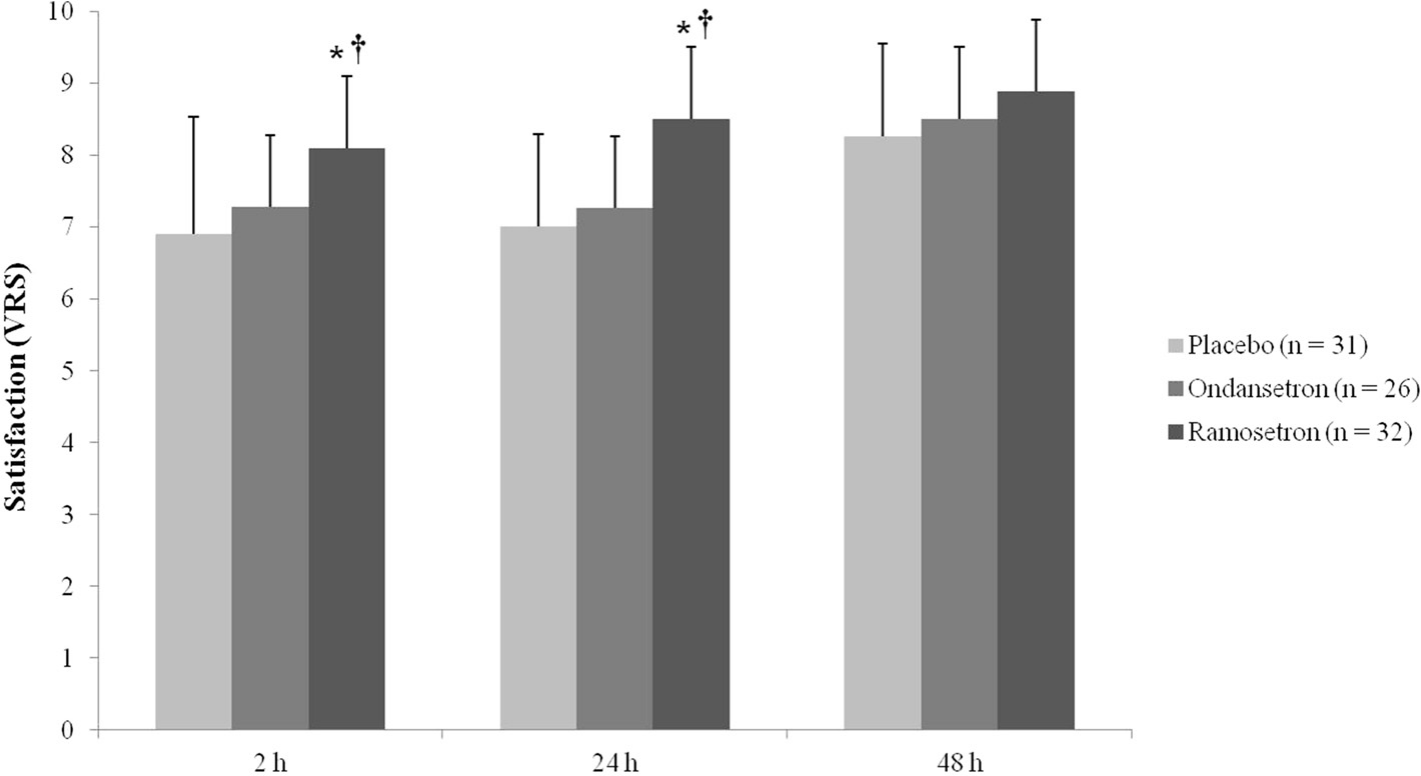
Patient satisfaction. VRS, verbal rating scale. *P < 0.05 versus placebo group, †*P* < 0.05 versus ondansetron group

Patients in the ramosetron group were more satisfied at 2 h (8.11 ± 0.98) and 24 h (8.50 ± 0.67) after surgery than those in the placebo (6.84 ± 1.34, 7.25 ± 1.29, respectively at 2 h and 24 h) and those in the ondansetron (7.28 ± 1.83, 7.27 ± 1.59, respectively at 2 h and 24 h) groups (*P* < 0.05). There was no significant difference in satisfaction in the patients at 48 h after surgery among any of the groups. The placebo and ondansetron groups showed no significant difference in patient satisfaction at all times after surgery (Fig. 3). There were no significant differences in the degree of pain and incidence of adverse effects of antiemetics among the groups (Tables 3 and 4).

**Table 3:** Degree of pain

	Placebo	Ondansetron	Ramosetron	*P* value
	(*n* = 31)	(*n* = 26)	(*n* = 32)	
VAS				
2 h	3.81 ± 1.87	3.35 ± 1.52	3.43 ± 1.74	ns
24 h	2.58 ± 1.52	2.08 ± 1.41	2.19 ± 1.31	ns
48 h	1.32 ± 1.40	1.58 ± 2.02	1.44 ± 1.19	ns

Data are means ± SD. *VAS* visual analogue scale

**Table 4:** Adverse effects of antiemetics

	Placebo	Ondansetron	Ramosetron	*P* value
	(*n* = 31)	(*n* = 26)	(*n* = 32)	
Headache				
2 h	7 (22.6)	3 (11.5)	9 (28.1)	ns
24 h	7 (22.6)	2 (7.7)	3 (9.4)	ns
48 h	1 (3.2)	0 (0)	0 (0)	ns
Dizziness				
2 h	7 (22.6)	4 (15.4)	4 (12.5)	ns
24 h	1 (3.2)	2 (7.7)	2 (6.3)	ns
48 h	0 (0)	0 (0)	0 (0)	ns
Drowsiness				
2 h	3 (9.7)	4 (15.4)	2 (6.3)	ns
24 h	0 (0)	0 (0)	1 (3.1)	ns
48 h	0 (0)	0 (0)	0 (0)	ns

Data are numbers (percentages)

**Fig. 4:**
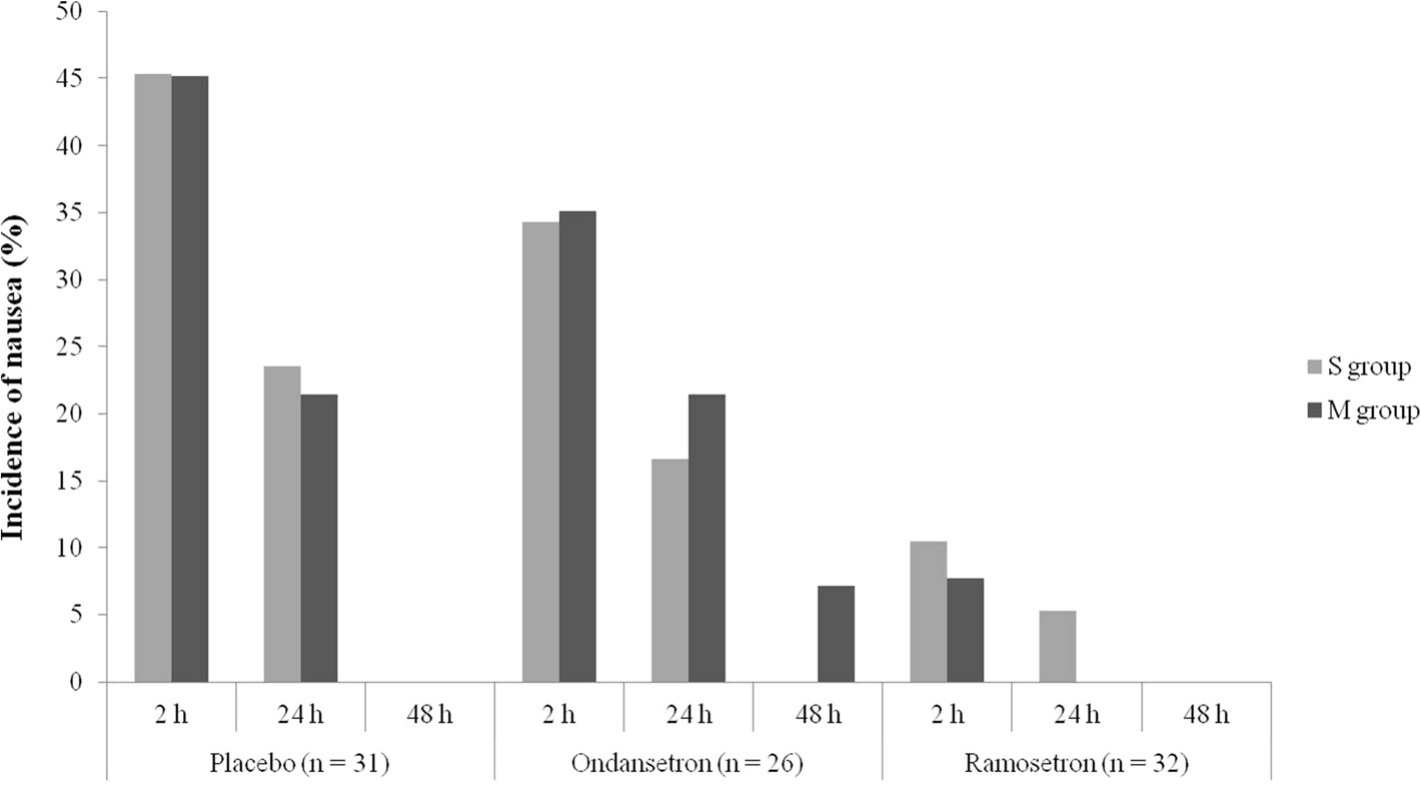
Incidence of nausea regarding the number of extraocular muscles involved Subgroup S, single-muscle correction; subgroup M, multiple-muscle correction. Placebo subgroup S (*n* = 16), subgroup M (*n* = 15); Ondansetron subgroup S (*n* = 12), subgroup M (*n* = 14); Ramoseron subgroup S (*n* = 17), subgroup M (*n* = 15)

**Fig. 5:**
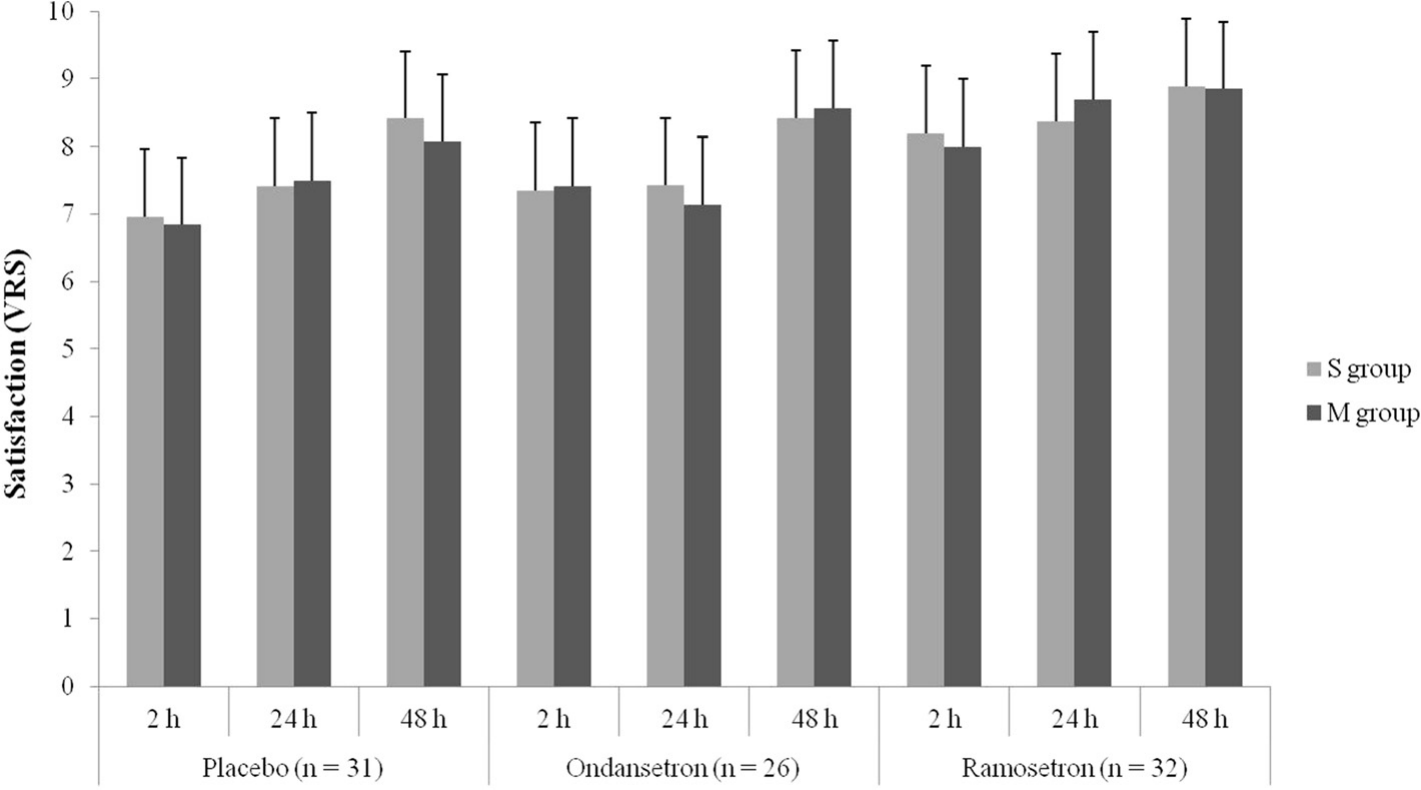
Patient satisfaction regarding the number of EOMs involved. Subgroup S, single-muscle correction; subgroup M, multiple-muscle correction. Placebo subgroup S (*n* = 16), subgroup M (*n* = 15); Ondansetron subgroup S (*n* = 12), subgroup M (*n* = 14); Ramoseron subgroup S (*n* = 17), subgroup M (*n* = 15). VRS, verbal rating scale

With regard to subgroups S and M in the placebo, ondansetron and ramosetron groups, there were no significant differences in either the incidence of nausea or patient satisfaction (Figs. 4 and 5). The ITT analysis also showed similar results.

## Discussion

PONV is one of the most common causes of unexpected hospitalization in ambulatory strabismus surgery [15]. Although the mechanism of PONV after strabismus surgery are not still clearly identified, traction of EOMs, besides from agents used for general anesthesia, is a well-known risk factor for PONV [11, 16, 17].

In the present study, the incidence of postoperative nausea without prophylaxis was 45.2 % at 2 h and 22.6 % at 24 h after strabismus surgery. This is slightly higher than the previously reported incidence of PONV after strabismus surgery [18–20]. Meanwhile, the incidence of postoperative vomiting without prophylaxis was 0 % in this study unlike those previous studies. However, analysis in these differences is complicated because of differences in study population and anesthetic techniques.

Drugs used to alleviate PONV act on serotonergic, dopaminergic, histaminergic, or cholinergic receptors in the chemoreceptor trigger zone [4, 21, 22]. Among these, the 5-HT_3_ receptor antagonists prevents the emetogenic signal from tranmission to the nucleus tractus solitarii, where most vagal afferents terminate, by regulating serotonin release. 5-HT_3_ receptor antagonists exert their effect by inhibiting the binding of serotonin to the 5-HT_3_ receptors; they are copious in nucleus tractus solitarii and the chemoreceptor trigger zone which, in turn, project signals to the emetic center located in the brainstem [23, 24].

Among 5-HT_3_ receptor antagonists, ondansetron was the first agent used clinically for PONV. A dose of 4 mg IV at the end of surgery has been reported to be effective for PONV prophylaxis, with a relatively short plasma half-life (3.5–4.7 h) and duration of action (~12 h) [13]. Tramer et al. [7] reported less pronounced antiemetic effect of ondansetron comparing to its anti-vomiting effect. Ondansetron, comparing with placebo, did not improve the patient’s satisfaction at any time point although it reduced the incidence of nausea only at 2 h after in our study.

Meanwhile, ramosetron, a newer 5-HT_3_ receptor antagonist, has greater affinity for 5-HT_3_ receptors and greater potency in consequence, and a longer plasma half life (5.8 ± 1.2 h) and duration of action than other 5-HT_3_ receptor antagonists [6, 8, 9]. Whether ramosetron has a greater antiemetic effect than other 5-HT_3_ receptor antagonists is controversial [25–30]. Our data suggest that ramosetron has a greater antiemetic effect than ondansetron within 24 h after strabismus surgery. Strabismus surgery is known to be a particularly emetogenic surgical procedure [1, 3, 31]. Improving patients’ satisfaction and stronger anti-emetic effect within 24 h after surgery comparing to ondansetron suggest that ramosetron should be administered after strabismus surgery to prevent nausea, because most postoperative nausea occurred within 24 h after surgery in our study population. Meanwhile, the antiemetic effect of ramosetron at 48 h after surgery was similar to that of ondansetron, which corresponds to the report of Halm et al. [10].

Among various ocular surgeries under general anesthesia, the strabismus surgery is one of the most common surgeries that would result in PONV. Traction of EOMs induces the ocluoemetic reflex and oculocardiac relex, causing bradycardia and hypotension, which are risk factors for PONV [11]. Therefore, we hypothesized that more PONV would occur as the number of EOMs involved in strabismus surgery increases, causing more muscle traction. However, this was not supported by our data. The effect of traction of EOMs on PONV after strabismus surgery still remains obscure. Further study is needed to reveal the mechanism of PONV after strabismus surgery.

Pain is a well-known risk factor for PONV, and nausea but not vomiting is the predominant symptom [11, 16]. As in the present study, several others have reported that adult patients complain of minimal pain after strabismus surgery with a VAS score < 3 [32, 33]. Moreover, the degree of pain did not differ among any of our groups. Therefore, we suggest that pain has less of an effect on PONV after strabismus surgery.

There are several limitations in our study. First, relatively small number of study population was included. Second, we did not discriminate the patients who had strabismus surgery in both eyes from those who had surgery in only one eye. Although we failed to demonstrate an association between the number of EOMs involved in strabismus surgery and PONV, the binocularity might have been related to PONV. Third, the equivalent doses of ramosetron and ondansetron has not been clearly identified. Various studies have compared the efficacy of 0.3 mg of ramosetron with 4–16 mg of ondansetron [9, 10, 24, 27, 34, 35]. In a previous animal study, ondansetron did not show a linear dose response curve [36], which indicates that it is quite complicated to decide the equivalent doses of ramosetron and ondanseston. Further investigation to verify the equivalent doses of ramosetron and ondansetron is in contemplation.

## Conclusions

In conclusion, the incidence of postoperative nausea was high until 24 h after strabismus surgery. Therefore, prevention of postoperative nausea during the 24 h after strabismus surgery is crucial. Ramosetron had an antiemetic efficacy greater than that of ondansetron or placebo during the first 24 h after strabismus surgery in adult patients. The number of EOMs involved in strabismus surgery was not associated with the incidence of PONV.

## Abbreviations

EOM, extraocular muscles; PONV, postoperative nausea and vomiting; TIVA, total intravenous anesthesia; VAS, visual analogue scale; VRS, verbal rating scale
